# Persistent recurring wheezing in the fifth year of life after laboratory-confirmed, medically attended respiratory syncytial virus infection in infancy

**DOI:** 10.1186/1471-2431-13-97

**Published:** 2013-06-19

**Authors:** Gabriel J Escobar, Anthony S Masaquel, Sherian X Li, Eileen M Walsh, Patricia Kipnis

**Affiliations:** 1 Division of Research, Perinatal Research Unit, Kaiser Permanente Medical Care Program, Oakland, CA, USA; 2 Health Outcomes and Pharmacoeconomics, MedImmune, LLC, Gaithersburg, MD, USA; 3 Management Information and Analysis, Kaiser Foundation Health Plan, Inc, Oakland, CA, USA

**Keywords:** Respiratory syncytial virus, Recurrent wheezing, Prematurity

## Abstract

**Background:**

Respiratory syncytial virus (RSV) infection in infancy is associated with subsequent recurrent wheezing.

**Methods:**

**A retrospective cohort study examined children born at** ≥**32 weeks gestation between 1996–2004.** All children were enrolled in an integrated health care delivery system in Northern California and were followed through the fifth year of life. The primary endpoint was recurrent wheezing in the fifth year of life and its association with laboratory-confirmed, medically-attended RSV infection during the first year, prematurity, and supplemental oxygen during birth hospitalization. Other outcomes measured were recurrent wheezing quantified through outpatient visits, inpatient hospital stays, and asthma prescriptions.

**Results:**

The study sample included 72,602 children. The rate of recurrent wheezing in the second year was 5.6% and fell to 4.7% by the fifth year. Recurrent wheezing rates varied by risk status: the rate was 12.5% among infants with RSV hospitalization, 8% among infants 32–33 weeks gestation, and 18% in infants with bronchopulmonary dysplasia. In multivariate analyses, increasing severity of respiratory syncytial virus infection was significantly associated with recurrent wheezing in year 5; compared with children without RSV infection in infancy, children who only had an outpatient RSV encounter had an adjusted odds ratio of 1.38 (95% CI,1.03–1.85), while children with a prolonged RSV hospitalization had an adjusted odds ratio of 2.59 (95% CI, 1.49–4.50).

**Conclusions:**

Laboratory-confirmed, medically attended RSV infection, prematurity, and neonatal exposure to supplemental oxygen have independent associations with development of recurrent wheezing in the fifth year of life.

## Background

Respiratory syncytial virus (RSV) infection is common during childhood; attack rates approach 100% by age 3 years [1]. Most infants have mild upper respiratory infections, but a significant proportion develop more severe lower respiratory tract disease [1]. The most important of these infections is bronchiolitis, and RSV is responsible for 50 to 80% of bronchiolitis hospitalizations and is the leading cause of hospitalization in children younger than 1 year in the United States [2-4].

A number of studies have reported an association between RSV infection and subsequent recurrent wheezing and early childhood asthma [5-12]. In particular, two recent reports have explored this relationship using large cohorts. Carroll et al. described the association between full-term children who had outpatient visits or hospitalizations for bronchiolitis in infancy and asthma in the sixth year of life [13]. They demonstrated the presence of a severity gradient (increased severity of bronchiolitis correlated with increased asthma severity). Using a more rigorous, laboratory-confirmed definition for RSV infection, our team previously reported a similar relationship between RSV infection and recurrent wheezing in the third year of life among both premature and full-term infants [14].

In this report, we describe the outcomes of a cohort of children born at ≥32 weeks gestational age (GA) who were followed through the fifth year of life. Because the association between RSV infection and asthma has been reported to decrease as the age of follow-up increases [5,6,15,16], we wanted to address this issue with an expanded cohort with two additional years of follow-up. Our goal was to quantify the relationship between laboratory-confirmed, medically attended RSV infection in the first year of life with the presence of recurrent wheezing (RW) during a time period when the prevalence of these conditions is expected to decrease [17]. We also explored the degree to which the relationships we found were dependent on the RW definition.

## Methods

### Study design

We collected data from Kaiser Permanente Northern California (KPNC), an integrated health care delivery system. This study was approved by the KPNC Institutional Review Board, which has jurisdiction over all the hospitals and clinics involved in this study. The inception cohort, described in detail in our previous report [14], included infants meeting the following criteria: birth at the KPNC Oakland, Hayward, Sacramento, San Francisco, Santa Clara, or Walnut Creek hospitals; date of birth from 1/1/96 through 12/31/04 (this study added infants born between 1/1/03 through 12/31/04; our previous cohort included births through 12/31/02); ≥32 weeks GA at birth; infant survived the birth hospitalization; and use of or membership in Kaiser Foundation Health Plan, Inc. during the first year of life.

### Study outcomes

Our primary study outcome was the occurrence of RW in the fifth year of life (fourth birthday through the day before the fifth birthday; henceforth, RW5). We defined RW as in our previous report using a combination of encounter events, patient diagnoses, and prescription patterns [14]. Using this definition, we determined whether children in our cohort met this definition in years 2, 3, 4, and 5 as well as examining the prevalence of any RW across years 2 through 5. We also tested a lenient definition termed “tentative asthma diagnosis” (TAD) in which we accepted any instance of International Classification of Diseases (ICD9) [18] diagnosis code for asthma (493.xx) in a given year at face value (i.e., we did not have strict criteria for number of encounters, nor did we use prescription data). Children categorized with TAD during years 2 through 5 were sorted into 4 hierarchical, mutually exclusive categories based on the year of life in which they were diagnosed.

### Predictors

The primary study predictor was the occurrence of laboratory-confirmed, medically attended RSV infection in the first year of life (henceforth, “RSV infection”). We also examined whether infants were infected with adenovirus, parainfluenza virus, influenza A or B virus, or *Bordetella pertussis*. We considered outpatient visits, emergency department (ED) visits, and inpatient hospitalization with diagnosis codes for each type of pathogen to be medically attended events. A medically attended event was considered to be laboratory-confirmed if there was a positive test for the pathogen within ±14 days of the encounter. Pathogens were identified using direct fluorescent antibody testing or culture. We categorized medically attended encounters for RSV and other pathogens as outpatient (including scheduled clinic visits, unscheduled urgent care visits, and ED visits), uncomplicated hospitalizations (hospitalizations with a total length of stay [LOS] <96 hours without assisted ventilation), or prolonged hospitalizations (hospitalizations lasting ≥96 hours or in which assisted ventilation occurred). Race was included as a predictor and assessed in this study because of the well-known differences in RSV infection severity by race. The other predictors — infant sex, maternal age, birth weight, GA, small for GA status, oxygen exposure during the neonatal period, family history of asthma as found in physicians’ Significant Problem List or because of parental visits for asthma, bronchopulmonary dysplasia, presence of congenital anomalies, number of siblings present in the home, and hospitalizations for unspecified respiratory illnesses — were defined as previously reported [14].

### Attrition

To be included in our main analyses, each child in the cohort had to be a member for at least 9 months in each of the first five years of life. To make this determination, we linked the study children’s utilization and membership data and created a monthly membership variable which was set equal to 1 (patient was a member during that month and/or had outpatient or inpatient use during that month) or 0 (patient was not a member during that month and did not have outpatient or inpatient use during that month) to determine eligibility for inclusion in the study cohort.

### Data collection

The KPNC information systems employ a common medical record number for all inpatient, outpatient, and administrative encounters in all facilities, permitting extraction and linkage of patient data using methods that have been previously described [19-22]. Care received outside the program was also tracked. We identified infants using the KPNC hospitalization database and the Neonatal Minimum Data Set [23], a database that captures supplemental oxygen use and assisted ventilation among infants receiving neonatal intensive care. These records were linked to patient demographic, membership, laboratory, pharmacy, outpatient encounter, and hospitalization databases to obtain the previously described variables.

### Statistical analyses

Statistical analyses were conducted using SAS (SAS Institute, Cary, NC). Unadjusted odds ratios (ORs) for all predictors were calculated using logistic regression [24] with RW5 as the outcome and each of the predictors as the only explanatory variable. Values of categorical variables were compared using a chi-square or Fisher exact test. Normally distributed continuous variables were compared using a Student *t* test. Comparisons of non-normally distributed continuous variables employed the Wilcoxon rank sum test.

We conducted multivariate analyses in three ways. We employed both logistic regression models in which children were included based on the abovementioned membership criteria as well as Cox proportional hazards modeling [24] (to account for censoring of infants). Because the results of Cox models were similar, we report those results in Additional file 1 Table S1; logistic regression models are reported in this paper. We also performed logistic regression using RW2, RW3, RW4, and RW5 cohorts so that we could assess the change in association as the length of follow-up increased.

The relative contribution of each predictor was calculated using the differences between the log likelihood of the full model and the log likelihood of a model without each of the predictors and was defined as the ratio of its log likelihood difference to the sum of the likelihood differences from all predictors × 100 [25].

## Results

### Study participants

Scanning KPNC databases identified 165,366 infants who survived to discharge from the birth hospitalization during 1996–2004. Infants were excluded from the study due to missing data (n=358), prematurity (<32 weeks GA [most received RSV immunoprophylaxis]; n=2,371) and not meeting membership criteria (n=19,913). The final study cohort consisted of 142,724 infants ≥32 weeks GA. Of these, 72,602 met membership criteria during the first 5 years of life and constitute the analysis cohort. Among these infants, 43,786, or 60.3%, were also in our original cohort. Additional details, including a cohort assembly flow chart, are available in Additional file 1 Figures S1 and S2.

Subject demographics are reported in Table 1. The study population is ethnically diverse, and the majority of mothers (73%) were 18–34 years of age. Using maternal records, we determined that 3.6% of infants had a maternal history of asthma.

**Table 1:** **Description of infants born during 1996**–**2004 by gestational age categories**

	**Gestational age, wk**					
	**32–33**	**34–36**	**37**	**38–40**	**≥ 41**	**All gestational ages**
N	936	4,665	4582	51,049	11,370	72,602
Male sex, %	51.07	54.81	53.40	50.44	50.71	50.96
Race, %*						
White	46.79	43.09	39.90	42.87	47.65	43.50
African-American	11.32	10.40	9.41	8.57	9.30	8.89
Asian	16.35	19.85	23.42	20.74	15.27	19.94
Hispanic	16.99	18.93	20.32	21.25	20.81	20.92
Other/unknown	8.55	7.74	6.96	6.56	6.97	6.75
Maternal age, y, %						
<18	2.24	1.29	1.18	1.19	1.49	1.25
18–34	67.20	68.75	70.76	73.58	74.64	73.17
>35	30.56	29.97	28.07	25.23	23.87	25.57
Family history of asthma, %^†^						
None	93.91	94.23	94.43	94.82	95.15	94.80
Father only	2.03	1.63	1.66	1.49	1.37	1.50
Mother only	4.06	4.14	3.84	3.60	3.42	3.63
Both parents	0.00	0.00	0.07	0.09	0.06	0.08
^3^1 sibling <5 y of age in home, %	42.95	42.17	39.57	39.51	32.22	38.58
Congenital anomaly present, %^‡^	13.14	10.33	7.77	6.52	6.24	6.88
Small for gestational age, %^§^	4.59	3.41	2.23	1.25	0.67	1.40
Oxygen exposure^||^ and BPD, %^¶^						
No oxygen, no BPD	55.77	86.13	96.49	97.77	96.31	96.17
<200 hours oxygen, no BPD	37.50	12.24	3.12	1.95	3.26	3.35
^3^200 hours oxygen, no BPD	5.24	1.29	0.37	0.25	0.40	0.41
BPD	1.50	0.34	0.02	0.03	0.04	0.07
Unspecified respiratory hospitalization %^#^	15.92	15.05	12.40	8.09	7.19	8.77
Laboratory-confirmed, medically attended RSV infection, %**	3.95	2.98	2.14	1.64	1.36	1.74
Admitted to hospital, %^‡‡^	2.46	1.31	0.87	0.63	0.49	0.69
Treated as outpatient, %^§§^	1.50	1.67	1.27	1.00	0.87	1.05
Laboratory-confirmed, medically attended infection with other pathogen, %^††^	0.64	0.58	0.31	0.28	0.25	0.30
Admitted to hospital, %^‡‡^	0.53	0.28	0.13	0.13	0.11	0.14
Treated as outpatient, %^§§^	0.11	0.3	0.17	0.16	0.14	0.16

Abbreviations: *BPD* Bronchopulmonary dysplasia, *CI* Confidence interval, *RSV* Respiratory syncytial virus, *wk* week, *y* year.

*Classification of race/ethnicity in this study was based on the race of infants' mothers, as self-reported to the birth certificate clerk interviewing them at the Kaiser Permanente hospitals described in this study. Race was assessed in this study because of the well-known differences in RSV infection severity by race.

^†^Ascertained by electronic scanning of parental records, which included encounters and diagnoses identified in the Significant Problem List by the parent’s physician.

^‡^See text for list of included International Classification of Diseases codes.

^§^Based on the algorithm of Brenner WE, et al. Am J Obstet Gynecol. 1976;126:555–564.

^||^Oxygen exposure, in hours, during the birth hospitalization. See text for details.

^¶^All infants with BPD had ≥200 hours of oxygen exposure during the neonatal period.

^#^Hospitalization for pneumonia or other respiratory illness without laboratory confirmation for RSV or the other pathogens listed in footnote ^††^.

**Medically attended visit where positive RSV direct fluorescent antibody test or viral culture occurred within ± 14 days of visit.

^††^Medically attended encounter where a positive test result (direct fluorescent antibody test, viral culture, or bacterial culture) for adenovirus, influenza virus, parainfluenza virus 1 or 2, or *Bordetella pertussis* occurred within ± 14 days of encounter.

^‡‡^ The encounter included a hospital admission.

^§§^ The encounter included outpatient care only.

### RSV infection and the prevalence of recurrent wheezing

Using logistic regression, documented RSV infection in the first year of life was associated with an increased risk of RW5, and a severity gradient was evident (Table 2). For example, RSV infection that only involved an outpatient encounter had an adjusted odds ratio (AOR) of 1.38 (95% CI, 1.03–1.85), whereas if it involved prolonged hospitalization the AOR was 2.59 (95% CI, 1.49–4.50). Exposure to oxygen in the neonatal period also showed a similar gradient. In contrast, infections with other pathogens were not associated with increased rates of RW5, although prolonged hospitalizations with unspecified organisms did show a strong association (AOR, 3.46, 95% CI, 1.94–6.16). Gestational age <37 weeks was also associated with increased risk of RW5 (Table 2). The relative contribution of RSV infection to the overall predictive ability of our model was 6.6%, whereas the relative contribution of oxygen exposure was 4.9%. In contrast, the relative contribution of non-modifiable risk factors was 22.8% for sex, 4.8% for GA, 21.2% for race, and 28.7% for family history of asthma.

**Table 2:** Risk factors for recurrent wheezing in the fifth year of life using logistic regression*

	**Beta**	**Adjusted odds ratio**	**95% CI**	***P *Value**
Sex				
Male	0.40	1.49	1.39–1.60	<.0001
Female	*reference*			
Race^†^				
White	*reference*			
African-American	0.53	1.71	1.52–1.91	<.0001
Asian	0.31	1.36	1.24–1.49	<.0001
Hispanic	0.10	1.11	1.01–1.22	0.0356
Other/unknown	0.22	1.25	1.09–1.44	0.0020
Maternal age, y				
<18	-0.07	0.94	0.69–1.28	0.6764
18–34	*reference*			
≥35	0.04	1.04	0.96–1.13	0.3321
Family history of asthma^†^				
Unknown	*reference*			
Father only	0.59	1.81	1.44–2.27	<.0001
Mother only	0.76	2.15	1.87–2.47	<.0001
Both parents	0.53	1.70	0.61–4.71	0.3109
Siblings <5 y of age in house				
None	*reference*			
≥1	-0.15	0.86	0.80–0.92	<.0001
Congenital anomaly^†^				
Present	0.13	1.14	1.00-1.29	0.0493
Absent	*reference*			
Small for gestational age^†^				
No	*reference*			
Yes	-0.04	0.96	0.72–1.28	0.7822
Gestational age, wk				
32–33	0.40	1.49	1.15–1.92	0.0022
34–36	0.26	1.30	1.14–1.48	<.0001
37	0.11	1.12	0.98–1.29	0.1049
38–40	*reference*			
≥41	-0.06	0.95	0.85–1.05	0.2717
Oxygen exposure and BPD^†^				
No oxygen exposure, no BPD	*reference*			
<200 hours oxygen, no BPD	0.28	1.32	1.12–1.57	0.0012
≥200 hours oxygen, no BPD	0.69	2.00	1.37–2.90	0.0003
BPD	1.03	2.80	1.31–5.98	0.0078
Unspecified respiratory hospitalization^†^				
None	*reference*			
Uncomplicated hospitalization	0.50	1.65	0.98–2.79	0.0594
Prolonged hospitalization	1.24	3.46	1.94–6.16	<.0001
Laboratory-confirmed, medically attended RSV infection^†^				
None	*reference*			
Outpatient visit only	0.32	1.38	1.03–1.85	0.0307
Uncomplicated hospitalization	0.68	1.98	1.39–2.81	0.0002
Prolonged hospitalization	0.95	2.59	1.49–4.50	0.0007
Laboratory-confirmed, medically attended infection with other pathogen^†^				
None	*reference*			
Outpatient visit only	0.26	1.30	0.62–0.69	0.4883
Uncomplicated hospitalization	0.32	1.38	0.54–3.52	0.4981
Prolonged hospitalization	0.74	2.10	0.76–5.80	0.1526

Abbreviations: *BPD* Bronchopulmonary dysplasia, *CI* Confidence interval, *RSV* Respiratory syncytial virus, *wk* week, *y* year.

*Based on study cohort of 72,602 children, of whom 3380 had recurrent wheezing in the fifth year of life. See text for description of cohort and definition of recurrent wheezing. Results have been adjusting for infant and mother demographic and clinical characteristics.

^†^Please refer to the appropriate section of the footnotes to Table 2 for further details.

^‡^Percent adherence to recommended course per AAP guidelines, see online appendix for details of how adherence was calculated.

**Figure 1:**
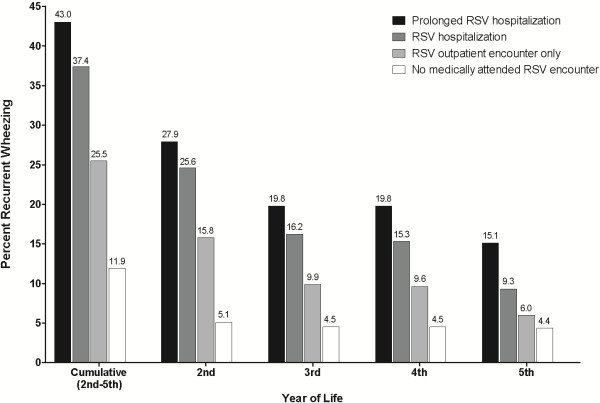
**Relationship between respiratory syncytial virus infection encounters in year 1 with prevalence of recurrent wheezing in years 2 through 5 of life.** Encounter types are hierarchical (prolonged hospitalization supersedes hospitalization, which supersedes outpatient encounter only) and mutually exclusive. RSV=respiratory syncytial virus.

The prevalence of RW by individual years and across years 2 through 5 is reported in Table 3. Across all children, the prevalence of RW decreased over time from 5.6% in year 2 to 4.7% in year 5. However, depending on the presence of risk factors, the rate of RW in year 2 and the rate of decline during early childhood may be substantially different. For example, 25.0% of children with laboratory-confirmed RSV hospitalization in their first year had RW in the second year of life. These infants were approximately three times as likely to have RW5 than children who did not (12.5% vs. 4.6%), and approximately three times as likely to ever have had RW in years 2–5 (40.0% vs 12.3%). Figure 1 shows that a severity gradient (increased severity of RSV infection in infancy is associated with an increased prevalence of RW) is evident across the second through fifth years of life. The association between RSV infection, RW, and prematurity is reported in Additional file 1 Figure S3, which provides additional displays of this gradient among children born at <37 and ≥37 weeks GA. In addition, the AOR for all types of RSV infection tended to fall as the dependent variable was changed from RW2 to RW5. For example, the AORs for an outpatient RSV encounter for RW2 through RW5 were 3.18, 2.21, 2.11, and 1.36. Additional results are shown in Additional file 1 Table S3.

**Table 3:** Recurrent wheezing during study period

		**RW 2nd Year**		**RW 3rd Year**		**RW 4th Year**		**RW 5th Year**		**RW 2nd–5th Years**	
	**N**	**n**	**%**	**n**	**%**	**n**	**%**	**n**	**%**	**n**	**%**
All children	72,602	4,091	5.6	3,492	4.8	3,466	4.8	3,380	4.7	9,151	12.6
Sex											
Female	35,606	1,402	3.9	1,261	3.5	1,318	3.7	1,330	3.7	3,499	9.8
Male	36,996	2,689	7.3	2,231	6.0	2,148	5.8	2,050	5.5	5,652	15.3
Race*											
White	31,580	1,650	5.2	1,443	4.6	1,353	4.3	1,265	4.0	3,642	11.5
African-American	6,454	517	8.0	409	6.3	420	6.5	441	6.8	1,093	16.9
Asian	14,476	807	5.6	774	5.3	785	5.4	764	5.3	1,983	13.7
Hispanic	15,189	876	5.8	644	4.2	670	4.4	664	4.4	1841	12.1
Other/unknown	4,903	241	4.9	222	4.5	238	4.9	246	5.0	592	12.1
Maternal age, y											
<18	911	55	6.0	38	4.2	37	4.1	44	4.8	118	13.0
18–34	53,125	3,068	5.8	2,530	4.8	2,571	4.8	2,453	4.6	6,779	12.8
≥35	18,566	968	5.2	924	5.0	858	4.6	883	4.8	2,254	12.1
Family history of asthma*											
None	68,824	3,698	5.4	3,131	4.5	3,135	4.6	3,053	4.4	8,320	12.1
Father only	1,087	91	8.4	94	8.6	92	8.5	83	7.6	220	20.2
Mother only	2,633	294	11.2	258	9.8	232	8.8	240	9.1	596	22.6
Both parents	58	8	13.8	9	15.5	7	12.1	4	6.9	15	25.9
Sibling aged <5 y in home											
None	44,590	2,358	5.3	2,127	4.8	2,246	5.0	2,197	4.9	5,631	12.6
≥1	28,012	1,733	6.2	1,365	4.9	1,220	4.4	1,183	4.2	3,520	12.6
Congenital anomaly*											
Absent	67,605	3,739	5.5	3,176	4.7	3,170	4.7	3,079	4.6	8,371	12.4
Present	4,997	352	7.0	316	6.3	296	5.9	301	6.0	780	15.6
Small for gestational age*											
No	71,582	4,011	5.6	3,424	4.8	3,405	4.8	3,328	4.6	8,989	12.6
Yes	1020	80	7.8	68	6.7	61	6.0	52	5.1	162	15.9
Gestational age, wk*											
32–33	936	99	10.6	81	8.7	78	8.3	75	8.0	188	20.1
34-36	4,665	401	8.6	290	6.2	294	6.3	294	6.3	793	17.0
37	4,582	270	5.9	264	5.8	209	4.6	238	5.2	635	13.9
38–40	51,049	2,763	5.4	2,373	4.6	2,345	4.6	2,290	4.5	6,235	12.2
≥41	11,370	558	4.9	484	4.3	540	4.7	483	4.2	1,300	11.4
Oxygen exposure* and BPD*											
No oxygen, no BPD	6,9821	3,819	5.5	3,267	4.7	3,249	4.7	3,162	4.5	8,607	12.3
<200 h Oxygen, no BPD	2,431	221	9.1	177	7.3	165	6.8	173	7.1	445	18.3
≥200 h Oxygen, no BPD	300	43	14.3	34	11.3	45	15.0	36	12.0	80	26.7
BPD	50	8	16.0	14	28.0	7	14.0	9	18.0	19	38.0
Unspecified respiratory hospitalization*											
None	72,345	4017	5.6	3,449	4.8	3,420	4.7	3,348	4.6	9,052	12.5
Uncomplicated hospitalization	177	47	26.6	24	13.6	26	14.7	16	9.0	64	36.2
Prolonged hospitalization	80	27	33.8	19	23.8	20	25.0	16	20.0	35	43.8
Laboratory-confirmed, medically attended RSV infection*											
None	71,336	3,829	5.4	3,326	4.7	3,312	4.6	3,280	4.6	8,748	12.3
Outpatient	762	127	16.7	79	10.4	74	9.7	50	6.6	201	26.4
Uncomplicated hospitalization	384	105	27.3	67	17.4	57	14.8	35	9.1	154	40.1
Prolonged hospitalization	120	30	25.0	20	16.7	23	19.2	15	12.5	48	40.0
Laboratory-confirmed, medically attended infection with other pathogen*											
None	72,382	4,056	5.6	3,476	4.8	3,449	4.8	3,362	4.6	9,101	12.6
Outpatient	119	20	16.8	7	5.9	7	5.9	8	6.7	30	25.2
Uncomplicated hospitalization	64	7	10.9	3	4.7	5	7.8	5	7.8	10	15.6
Prolonged hospitalization	37	8	21.6	6	16.2	5	13.5	5	13.5	10	27.0
Definite, medically attended RSV encounter in the first year of life*											
No	71,336	3,829	5.4	3,326	4.7	3,312	4.6	3,280	4.6	8,748	12.3
Yes	1,266	262	20.7	166	13.1	154	12.2	100	7.9	403	31.8

Abbreviations: *BPD* Bronchopulmonary dysplasia, *RSV*  Respiratory syncytial virus, *RW* Recurrent wheezing, *wk* weeks, *y* years.

*Please refer to the appropriate section of the footnotes to Table 1 for further details.

Use of a more lenient tentative asthma diagnosis to capture RW events did not lead to any directional change in our findings, but rather a general increase in the prevalence of reported outcomes. The proportion of children with RW5 using our strict definition was 4.6%, but increased to 10.3% when using the more lenient definition; however, the overall trends in the data remained consistent. Tabular data using this alternative definition is shown in Additional file 1 Table S2 analyses using this definition are available upon request by interested readers.

### Recurrent wheezing in preterm and term infants

We quantified the occurrence of any RW episode (outpatient or inpatient encounter with ICD diagnosis 493.xx) across all study years in term and premature infants. This approach demonstrated higher rates of RW encounters among infants with RSV infection compared with infants without RSV infection and also a gradient by severity of RSV infection. Approximately 30%–60% of premature infants 32–36 weeks GA with RSV infection during the first year of life will have a recurrent wheezing episode by age 5 years and 40% will have >1 episode. The overall rate of inpatient encounters with a wheezing diagnosis among premature infants without RSV infections in infancy was 56 per 1,000 children by age 5 years, whereas the rate among those that did have RSV infection was 102 per 1,000 children; the corresponding rates for term infants were 27 and 111, respectively. With respect to outpatient encounters, the respective rates were 1,097 and 2,352 per 1,000 children for premature infants and 743 and 2,152 for term infants.

## Discussion

In this retrospective cohort study of children born at ≥32 weeks GA, we found that three important risk factors (RSV disease in the first year of life, moderate prematurity, and exposure to supplemental oxygen in the neonatal period) had statistically significant associations with the development of RW5. This is consistent with our previous report, [14] where these predictors were associated with RW in the third year of life. Our study is concordant with and expands upon the study by Carroll et al. [13]. As was the case with that study, ours is a very large cohort, but ours had the advantage of including children born at <37 weeks gestation as well as actually documenting RSV infection, rather than just using the presence of a bronchiolitis diagnosis. Our study also found that other pathogens did not show a strong association. We also found that, consistent with the known epidemiology of asthma and RW, prevalence decreases over time, but among infants with certain risk factors including RSV infection, the prevalence of RW remained high in the fifth year. We found that these relationships persisted when we employed a more lenient definition of RW (accepting any instance of an asthma diagnosis). Future studies may be needed to better characterize the burden of these infants on the healthcare system and determine whether other strategies for mitigating the effects of RSV are possible. It is important to stress that our study is not in concordance with another very large study conducted in Finland, where Dunder et al. did not find an association between RSV epidemics and subsequent use of asthma medication [26]. The study by Dunder et al. is important because, like us, electronic scanning of asthma medications was employed for ascertainment.

In our previous report [14], we pointed out a number of limitations to our findings, of which the most important was that not all infants in our cohort were tested for RSV. Nonetheless, the presence of a severity gradient, the fact that the RSV effect observed in the third year of life persists into the fifth year, and the fact that other pathogens do not show this association, strongly support the notion that RSV infection in infancy is associated with asthma in childhood. This notion has been suggested by others [5-9]. However, it is important to note that some of our findings could be interpreted differently. For example, while it is true that our analyses clearly show a specific effect of documented RSV infection, it is striking that the relative contribution of this variable to the overall predictive ability of our model is small (6.6%), while the contribution of non-modifiable risk factors such as sex, race, and family history was far greater. In this context, we must note that our lack of data on tobacco exposure is a significant limitation.

The presence of an association, however strong, does not constitute evidence of causality, and it is important to consider a number of possible causal paths, which are not mutually exclusive, that could explain our findings. The relationship of lower respiratory tract RSV disease to asthma is under investigation, particularly the role of genetic susceptibility for eventual asthma and the interaction of genetic susceptibility with the environment [27-29]. It remains unclear whether RSV infection disrupts neural control of bronchiolar smooth muscle setting the stage for sensitized airways or whether RSV disease serves as an additional insult to a host with pre-existing genetic susceptibility for eventual asthma [27,30-33]. One possibility is that infection with RSV causes lung damage that ultimately leads to the development of asthma. Such damage could be accentuated by oxygen exposure, prematurity, bronchopulmonary dysplasia, and/or environmental exposures (e.g., air pollution, secondhand smoke). Another possibility is that a set of genes exists that is preferentially expressed following infection with RSV. Such genes, which could also be affected by environmental factors, could lead to the occurrence of wheezing and asthma. It is also possible that a set of genes that are not identical to, but are related to, the development of asthma also code for a more exaggerated response to RSV infection, thus explaining the severity gradient noted by us and others. Lastly, one cannot exclude the possibility that a subset of the population requires a severe RSV infection in order to develop asthma.

Given conflicting findings in the literature, with different studies and study types (e.g., twin studies) coming to different conclusions [28,34,35], a definitive explanation of the relationship of RSV infection to the development of asthma remains elusive [36]. What is clear is that future studies need to include more detail with respect to direct capture of genetic information. It is instructive to outline the components of what could be considered an optimal prospective study to explore the relationship between RSV infection in infancy and the development of asthma. Ideally, infants should be enrolled immediately after birth, and the initial intake process should include non-invasive collection of infant and parental DNA and a detailed history that includes special attention to family smoking history, history of atopy in parents and grandparents, and other possible environmental and allergen exposures (e.g., pets in the home). Subsequent follow-up should include frequent testing for respiratory pathogens [37], careful attention to the development of atopy, and an objective assessment of whether or not a child develops wheezing. The setting for such a study should be in an ethnically diverse population with a relatively high baseline prevalence of asthma.

## Conclusions

We have found that predictors for recurrent wheezing in the third year of life also predict for recurrent wheezing in the fifth year. Of these, the most important potentially modifiable predictor was documented laboratory-confirmed, medically attended RSV infection in the first year of life. Future research in this area will need to incorporate genetic data collection, since purely observational studies do not permit definitive inferences as to causal pathways responsible for the association between RSV infection and subsequent wheezing.

## Abbreviations

KPNC: Kaiser Permanente Northern California; RW: Recurrent wheezing; RW3: Recurrent wheezing at age 3 years; RW5: Recurrent wheezing at age 5 years; RSV: Respiratory syncytial virus.

## Competing interests

At the time of this study, Dr. Masaquel was a MedImmune employee and received an incentive plan. The other authors have no conflict of interest.

## Authors’ contributions

GJE was the principal investigator, had full access to all data in the study and supervised data collection, data cleaning, and, in conjunction with PK, the project statistician, supervised all project analyses. ASM was involved with the design of the study, interpretation of the data, and review of the manuscript. All authors agreed on the final text and conclusions of the manuscript.

## Pre-publication history

The pre-publication history for this paper can be accessed here:

http://www.biomedcentral.com/1471-2431/13/97/prepub

## Supplementary Material

Additional file 1 Supplementary information. Click here for file
