# Supplementary material for: Persistent recurring wheezing in the fifth year of life after laboratory-confirmed, medically attended respiratory syncytial virus infection in infancy
Source: BMC Pediatr. 2013 Jun 19;13:97. doi: 10.1186/1471-2431-13-97 (PMC3703269; doi:10.1186/1471-2431-13-97)
Supplement: Additional file 1 — Supplementary information. [file 1471-2431-13-97-S1.doc]

**Supplementary Table 1. Cox proportional hazards model for recurrent wheezing over time.**

|  | **Beta** | **Hazard Ratio** | **95% CI** | | | ***P* Value** |
| --- | --- | --- | --- | --- | --- | --- |
| Sex |  |  |  |  |  |  |
| Male | 0.37 | 1.45 | 1.37–1.54 | | | <.0001 |
| Female | reference |  |  |  |  |  |
| Race* |  |  |  |  |  |  |
| White | reference |  |  |  |  |  |
| African-American | 0.53 | 1.69 | 1.54–1.86 | | | <.0001 |
| Asian | 0.34 | 1.40 | 1.30–1.51 | | | <.0001 |
| Hispanic | 0.13 | 1.14 | 1.05–1.23 | | | 0.0011 |
| Other/unknown | 0.15 | 1.16 | 1.04–1.29 | | | 0.0101 |
| Maternal age, y |  |  |  |  |  |  |
| <18 | -0.06 | 0.94 | 0.73–1.20 | | | 0.6140 |
| 18–34 | reference |  |  |  |  |  |
| ≥35 | 0.10 | 1.11 | 1.04–1.18 | | | 0.0022 |
| Family history of asthma^†^ |  |  |  |  |  |  |
| Unknown | reference |  |  |  |  |  |
| Father only | 0.67 | 1.95 | 1.63–2.33 | | | <.0001 |
| Mother only | 0.78 | 2.19 | 1.97–2.44 | | | <.0001 |
| Both parents | 0.59 | 1.81 | 0.86–3.81 | | | 0.1160 |
| <5 yrs Siblings in house |  |  |  |  |  |  |
| None | reference |  |  |  |  |  |
| ≥1 | –0.14 | 0.87 | 0.82–0.92 | | | <.0001 |
| Congenital anomaly present^‡^ |  |  |  |  |  |  |
| No | reference |  |  |  |  |  |
| Yes | 0.14 | 1.15 | 1.03–1.27 | | | 0.0098 |
| Small for gestational age^§^ |  |  |  |  |  |  |
| No | reference |  |  |  |  |  |
| Yes | –0.08 | 0.92 | 0.73–1.16 | | | 0.4959 |
| Gestational age at birth, wk |  |  |  |  |  |  |
| 32–33 | 0.42 | 1.53 | 1.26–1.86 | | | <.0001 |
| 34–36 | 0.18 | 1.19 | 1.07–1.33 | | | 0.0011 |
| 37 | 0.09 | 1.10 | 0.98–1.23 | | | 0.1040 |
| 38–40 | reference |  |  |  |  |  |
| 41+ | –0.09 | 0.92 | 0.84–1.00 | | | 0.0435 |
| Oxygen exposure** and BPD^††^ |  |  |  |  |  |  |
| No oxygen exposure, no BPD | reference |  |  |  |  |  |
| < 200 hours oxygen, no BPD | 0.29 | 1.34 | 1.17–1.54 | | | <.0001 |
| 200 hours oxygen, no BPD | 0.67 | 1.95 | 1.45–2.64 | | | <.0001 |
| BPD | 0.92 | 2.50 | 1.43–4.40 | | | 0.0014 |
| Unspecified respiratory hospitalization^‡‡^ |  |  |  |  |  |  |
| None | reference |  |  |  |  |  |
| Uncomplicated hospitalization | 0.77 | 2.15 | 1.52–3.05 | | | <.0001 |
| Prolonged hospitalization | 1.06 | 2.88 | 1.90–4.37 | | | <.0001 |
| Laboratory-confirmed, medically attended RSV infection^§§^ |  |  |  |  |  |  |
| None | reference |  |  |  |  |  |
| Outpatient visit only | 0.43 | 1.54 | 1.24–1.92 | | | <.0001 |
| Uncomplicated hospitalization | 0.74 | 2.10 | 1.61–2.73 | | | <.0001 |
| Prolonged hospitalization | 0.79 | 2.21 | 1.44–3.41 | | | 0.0003 |
| Laboratory-confirmed, medically attended infection with other pathogen*** |  |  |  |  |  |  |
| None | reference |  |  |  |  |  |
| Outpatient encounter only | 0.28 | 1.32 | 0.78–2.24 | | | 0.2954 |
| Uncomplicated hospitalization | 0.74 | 2.11 | 1.16–3.81 | | | 0.0138 |
| Prolonged hospitalization | 0.35 | 1.41 | 0.58–3.43 | | | 0.4448 |

BPD = bronchopulmonary dysplasia; CI = confidence interval; RSV = respiratory syncytial virus; y = year; wk = week. Total number of observations is 142724, of which 4826 have outcome.

*Classification of race/ethnicity based on the race of infants' mothers, as self-reported to the birth certificate clerk interviewing them at the Kaiser Permanente hospitals described in this study. Race was assessed in this study because of the well-known differences in RSV severity by race.

^†^Ascertained by electronic scanning of parental records, which included encounters and diagnoses identified in the Significant Problem List by the parent’s physician.

^‡^See article text for list of included International Classification of Diseases codes

^§^Based on the algorithm of Brenner WE, et al. Am J Obstet Gynecol. 1976; 126:555-564.

**Oxygen exposure, in hours, during the birth hospitalization. See text for details.

^††^All infants with BPD has ≥200 hours of oxygen exposure during the neonatal period.

^‡‡^Hospitalization for pneumonia or other respiratory illness without laboratory confirmation for RSV or other pathogens listed

^§§^ Medically attended visit where positive RSV direct fluorescent antibody test or viral culture occurred within ±14 days of visit.

***Medically attended encounter where a positive test result (direct fluorescent antibody test, viral culture, or bacterial culture) for adenovirus, influenza virus, parainfluenza virus 1 or 2, or *Bordetella pertussis* occurred within ±14 days of encounter.

**Supplementary Table 2. Tentative asthma diagnosis leniently defined, over study period.**

|  |  | **TAD 2nd**  **Year** | | **TAD 3rd Year** | | **TAD 4th Year** | | **TAD 5th Year** | | **TAD 2nd–5th Year** | |
| --- | --- | --- | --- | --- | --- | --- | --- | --- | --- | --- | --- |
|  | **N** | n | % | n | % | n | % | n | % | n | % |
| All children | 72602 | 7340 | 10.1 | 6856 | 9.4 | 7313 | 10.1 | 7613 | 10.5 | 16441 | 22.6 |
| Sex |  |  |  |  |  |  |  |  |  |  |  |
| Female | 35606 | 2734 | 7.7 | 2595 | 7.3 | 2878 | 8.1 | 3088 | 8.7 | 6636 | 18.6 |
| Male | 36996 | 4606 | 12.5 | 4261 | 11.5 | 4435 | 12.0 | 4525 | 12.2 | 9805 | 26.5 |
| Race^†^ |  |  |  |  |  |  |  |  |  |  |  |
| White | 31580 | 2894 | 9.2 | 2734 | 8.7 | 2803 | 8.9 | 2901 | 9.2 | 6543 | 20.7 |
| African-American | 6454 | 952 | 14.8 | 867 | 13.4 | 927 | 14.4 | 955 | 14.8 | 1952 | 30.2 |
| Asian | 14476 | 1419 | 9.8 | 1445 | 10.0 | 1605 | 11.1 | 1682 | 11.6 | 3462 | 23.9 |
| Hispanic | 15189 | 1622 | 10.7 | 1367 | 9.0 | 1484 | 9.8 | 1537 | 10.1 | 3384 | 22.3 |
| Other/unknown | 4903 | 453 | 9.2 | 443 | 9.0 | 494 | 10.1 | 538 | 11.0 | 1100 | 22.4 |
| Maternal age, y |  |  |  |  |  |  |  |  |  |  |  |
| <18 | 911 | 132 | 14.5 | 108 | 11.9 | 102 | 11.2 | 102 | 11.2 | 239 | 26.2 |
| 18–34 | 53125 | 5517 | 10.4 | 5100 | 9.6 | 5395 | 10.2 | 5582 | 10.5 | 12221 | 23.0 |
| ≥35 | 18566 | 1691 | 9.1 | 1648 | 8.9 | 1816 | 9.8 | 1929 | 10.4 | 3981 | 21.4 |
| Family History of asthma^†^ |  |  |  |  |  |  |  |  |  |  |  |
| None | 68824 | 6672 | 9.7 | 6200 | 9.0 | 6656 | 9.7 | 6938 | 10.1 | 15055 | 21.9 |
| Father only | 1087 | 152 | 14.0 | 167 | 15.4 | 167 | 15.4 | 174 | 16.0 | 345 | 31.7 |
| Mother only | 2633 | 504 | 19.1 | 476 | 18.1 | 476 | 18.1 | 487 | 18.5 | 1017 | 38.6 |
| Both parents | 58 | 12 | 20.7 | 13 | 22.4 | 14 | 24.1 | 14 | 24.1 | 24 | 41.4 |
| Sibling aged <5 y in home |  |  |  |  |  |  |  |  |  |  |  |
| None | 44590 | 4230 | 9.5 | 4126 | 9.3 | 4655 | 10.4 | 4867 | 10.9 | 10118 | 22.7 |
| ≥1 | 28012 | 3110 | 11.1 | 2730 | 9.7 | 2658 | 9.5 | 2746 | 9.8 | 6323 | 22.6 |
| Congenital anomaly^†^ |  |  |  |  |  |  |  |  |  |  |  |
| Absent | 67605 | 6700 | 9.9 | 6284 | 9.3 | 6715 | 9.9 | 6938 | 10.3 | 15079 | 22.3 |
| Present | 4997 | 640 | 12.8 | 572 | 11.4 | 598 | 12.0 | 675 | 13.5 | 1362 | 27.3 |
| Small for gestational age^†^ |  |  |  |  |  |  |  |  |  |  |  |
| No | 71582 | 7208 | 10.1 | 6744 | 9.4 | 7186 | 10.0 | 7496 | 10.5 | 16163 | 22.6 |
| Yes | 1020 | 132 | 12.9 | 112 | 11.0 | 127 | 12.5 | 117 | 11.5 | 278 | 27.3 |
| Gestational age, wk |  |  |  |  |  |  |  |  |  |  |  |
| 32–33 | 936 | 165 | 17.6 | 145 | 15.5 | 150 | 16.0 | 143 | 15.3 | 310 | 33.1 |
| 34–36 | 4665 | 642 | 13.8 | 562 | 12.0 | 612 | 13.1 | 626 | 13.4 | 1327 | 28.4 |
| 37 | 4582 | 510 | 11.1 | 491 | 10.7 | 481 | 10.5 | 545 | 11.9 | 1157 | 25.3 |
| 38–40 | 51049 | 4999 | 9.8 | 4690 | 9.2 | 4996 | 9.8 | 5177 | 10.1 | 11274 | 22.1 |
| ≥41 | 11370 | 1024 | 9.0 | 968 | 8.5 | 1074 | 9.4 | 1122 | 9.9 | 2373 | 20.9 |
| Oxygen exposure^†^ and BPD^†^ |  |  |  |  |  |  |  |  |  |  |  |
| No oxygen, no BPD | 69821 | 6884 | 9.9 | 6467 | 9.3 | 6886 | 9.9 | 7192 | 10.3 | 15554 | 22.3 |
| <200 h Oxygen, no BPD | 2431 | 373 | 15.3 | 320 | 13.2 | 345 | 14.2 | 340 | 14.0 | 740 | 30.4 |
| ≥200 h Oxygen, no BPD | 300 | 73 | 24.3 | 54 | 18.0 | 71 | 23.7 | 67 | 22.3 | 127 | 42.3 |
| BPD | 50 | 10 | 20.0 | 15 | 30.0 | 11 | 22.0 | 14 | 28.0 | 20 | 40.0 |
| Unspecified respiratory hospitalization^†^ |  |  |  |  |  |  |  |  |  |  |  |
| None | 72345 | 7228 | 10.0 | 6783 | 9.4 | 7239 | 10.0 | 7555 | 10.4 | 16294 | 22.5 |
| Uncomplicated hospitalization | 177 | 77 | 43.5 | 45 | 25.4 | 49 | 27.7 | 38 | 21.5 | 100 | 56.5 |
| Prolonged hospitalization | 80 | 35 | 43.8 | 28 | 35.0 | 25 | 31.3 | 20 | 25.0 | 47 | 58.8 |
| Laboratory-confirmed, medically attended RSV infection^†^ |  |  |  |  |  |  |  |  |  |  |  |
| None | 71336 | 6940 | 9.7 | 6554 | 9.2 | 7022 | 9.8 | 7370 | 10.3 | 15845 | 22.2 |
| Outpatient | 762 | 195 | 25.6 | 148 | 19.4 | 150 | 19.7 | 132 | 17.3 | 316 | 41.5 |
| Uncomplicated hospitalization | 384 | 155 | 40.4 | 113 | 29.4 | 108 | 28.1 | 81 | 21.1 | 213 | 55.5 |
| Prolonged hospitalization | 120 | 50 | 41.7 | 41 | 34.2 | 33 | 27.5 | 30 | 25.0 | 67 | 55.8 |
| Laboratory-confirmed, medically attended infection with other pathogen^†^ |  |  |  |  |  |  |  |  |  |  |  |
| None | 72382 | 7282 | 10.1 | 6820 | 9.4 | 7270 | 10.0 | 7572 | 10.5 | 16354 | 22.6 |
| Outpatient | 119 | 30 | 25.2 | 17 | 14.3 | 23 | 19.3 | 24 | 20.2 | 46 | 38.7 |
| Uncomplicated hospitalization | 64 | 16 | 25.0 | 9 | 14.1 | 10 | 15.6 | 10 | 15.6 | 22 | 34.4 |
| Prolonged hospitalization | 37 | 12 | 32.4 | 10 | 27.0 | 10 | 27.0 | 7 | 18.9 | 19 | 51.4 |
| Definite, medically attended RSV encounter in first year |  |  |  |  |  |  |  |  |  |  |  |
| No | 71336 | 6940 | 9.7 | 6554 | 9.2 | 7022 | 9.8 | 7370 | 10.3 | 15845 | 22.2 |
| Yes | 1266 | 400 | 31.6 | 302 | 23.9 | 291 | 23.0 | 243 | 19.2 | 596 | 47.1 |

BPD=bronchopulmonary dysplasia; RSV=respiratory syncytial virus; TAD=tentative asthma diagnosis; wk=week; y=year.

**^†^**Please refer to the appropriate section of the footnotes to Supplementary Table 1 for further details.

Subjects were classified as having TAD if there was any instance of ICD-9 code 493.xx (with no criteria for number or types of encounters, nor for prescriptions for asthma medications) in their medical records during the study period. We categorized TAD similarly to the way we categorized RW, determining whether the children in our cohort met this definition in years 2, 3, 4, and 5, whether they experienced any TAD during years 2 through 5, and by sorting children into 4 hierarchical, mutually exclusive categories, as described.

**Supplementary Table 3. Logistic regression models focusing on RSV effect on recurrent wheezing in years 2 through 5.**

|  | **Beta** | **Odds Ratio** | **95% CI** | **p value** |
| --- | --- | --- | --- | --- |
| Year 5^*^ |  |  |  |  |
| RSV infection |  |  |  |  |
| None | Ref | -- | -- | -- |
| Outpatient encounter only | 0.32 | 1.38 | 1.03-1.85 | 0.0307 |
| Uncomplicated hospitalization | 0.68 | 1.98 | 1.39-2.81 | 0.0002 |
| Prolonged hospitalization | 0.95 | 2.59 | 1.49-4.50 | 0.0007 |
|  |  |  |  |  |
| Year 4^†^ |  |  |  |  |
| RSV infection |  |  |  |  |
| None | Ref | -- | -- | -- |
| Outpatient encounter only | 0.75 | 2.11 | 1.65-2.70 | <.0001 |
| Uncomplicated hospitalization | 1.23 | 3.44 | 2.58-4.59 | <.0001 |
| Prolonged hospitalization | 1.48 | 4.39 | 2.75-7.00 | <.0001 |
|  |  |  |  |  |
| Year 3**^‡^** |  |  |  |  |
| RSV infection |  |  |  |  |
| None | Ref | -- | -- | -- |
| Outpatient encounter only | 0.79 | 2.21 | 1.74-2.81 | <.0001 |
| Uncomplicated hospitalization | 1.38 | 3.98 | 3.04-5.22 | <.0001 |
| Prolonged hospitalization | 1.24 | 3.46 | 2.11-5.67 | <.0001 |
|  |  |  |  |  |
| Year 2^§^ |  |  |  |  |
| RSV infection |  |  |  |  |
| None | Ref | -- | -- | -- |
| Outpatient encounter only | 1.16 | 3.18 | 2.61-3.88 | <.0001 |
| Uncomplicated hospitalization | 1.78 | 5.96 | 4.72-7.52 | <.0001 |
| Prolonged hospitalization | 1.56 | 4.75 | 3.10-7.29 | <.0001 |

Models are adjusted for all other covariates, which are listed in Table 1 of the main manuscript.

^*^ The total number of observations is 72602, of which 3380 have the outcome.

^†^ The total number of observations is 72602, of which 3466 have the outcome.

^‡^ The total number of observations is 72602, of which 3492 have the outcome.

^§^ The total number of observations is 72602, of which 4091 have the outcome.

**Supplementary Figure 1: Cohort assembly.**

**Supplementary Figure 2. Overlap between the original (RW3) study cohort and this (RW5) cohort.**

**Supplementary Figure 3. Relationship between respiratory syncytial virus infection encounters in year 1 with prevalence of recurrent wheezing in years 2 through 5 of life among infants born at ≥37 weeks gestation (A) and born at 32–36 weeks gestation (B).** Encounter types are hierarchical (prolonged hospitalization supersedes hospitalization, which supersedes outpatient encounter only) and mutually exclusive. RSV=respiratory syncytial virus.
